# Validity and reliability of the Johns Hopkins Adapted Cognitive Exam for critically ill patients

**DOI:** 10.1186/cc9782

**Published:** 2011-03-11

**Authors:** MM Mirski, JL Lewin, SL Ledroux, KS Shermock, CT Thompson, HG Goodwin, EM Mirski, RG Gill

**Affiliations:** 1 Johns Hopkins Medicine, Baltimore, MD, USA

## Introduction

Assessment of cognition in ICU patients is a critical component of evaluating cerebral dysfunction. Several cognitive tools also exist for assessment of delirium in the ICU. However, few are simple to use and none has been specifically designed to focus on cognition in ICU patients. The Johns Hopkins Adapted Cognitive Exam (ACE) is an examination tool on a 100-point scale specifically designed for the assessment and quantification of cognition in critically ill patients.

## Methods

A prospective cohort study to establish the criterion, construct, and face validity, as well as inter-rater reliability and inter-item reliability of the ACE.

## Results

A total of 106 patients were assessed, 46 intubated and 60 non-intubated, resulting in 424 ACE measurements and 240 MMSE measurements. ACE and MMSE were performed by 76 different raters over the study period. For criterion validity we compared ACE with a neurointensivist's assessment of cognitive status (*r*_s _= 0.83, *P *< 0.001). In addition we utilized an ordinal logistic regression model to establish optimal predicted cut-off points for cognitive status classification (< 28 = severely impaired, 29 to 55 = moderately impaired, >56 = mildly impaired or normal). Utilizing these cut-off points, the ACE appropriately classified cognitive status 90% of the time as compared with the neurointensivist assessment. Construct validity was established by comparing ACE with MMSE in non-intubated patients (*r*_s _= 0.81, *P *< 0.001). Face validity was assessed by surveying raters who used both the ACE and MMSE during the study, and indicated the ACE was an accurate reflection of the patient's cognitive status, was more sensitive a marker of cognition than the MMSE, and was easy to use. The ACE demonstrated excellent inter-rater reliability (ICC = 0.997, 95% CI = 0.997 to 0.998). In addition, inter-item reliability of each of the five subscales of the ACE and MMSE was also assessed (Cronbach's alpha: range for ACE = 0.83 to 0.88; range for MMSE = 0.72 to 0.81), demonstrating a higher degree of internal consistency across subscales for the ACE.

## Conclusions

The ACE is the first valid and reliable examination for the assessment and quantification of cognition in critically ill patients. It provides a useful, objective tool that can be utilized by any member of the interdisciplinary critical care team to support clinical assessment and research efforts.
